# Third harmonic generation microscopy reveals structure and mucus dynamics in human airway epithelium models

**DOI:** 10.1016/j.isci.2026.117547

**Published:** 2026-09-25

**Authors:** Doyoung Kim, Alexandra Latshaw, Marta Balkota, Moritz Wiggert, Milvia Alata, Song Huang, Samuel Constant, Pierre Maechler, Pieter Vanden Berghe, Luigi Bonacina

**Affiliations:** 1 Department of Applied Physics, Université de Genève, Rue de l’Ecole-de-Médecine 20, 1205 Genève, Switzerland; 2 Department of Cell Physiology and Metabolism, Université de Genève, Rue Michel-Servet 1, 1206 Genève, Switzerland; 3 Laboratory of Enteric Neuroscience (LENS), Translational Research in Gastrointestinal Disorders (TARGID), CHROMETA, University of Leuven, Leuven, Belgium; 4 Epithelix Sàrl, Chemin des Aulx 18, Plan-les-Ouates, Genève 1228, Switzerland

**Keywords:** third harmonic generation, airway epithelium, air-liquid interface, ALI, cultures, mucociliary transport

## Abstract

Airway epithelium is the primary interface between human body and the external environment, acting as a physical and functional barrier. *In vitro* airway models that reproduce the epithelium architecture are valuable tools for studying infection, inflammation, and transport processes. In this work, we present a label-free, non-invasive method to visualize and measure mucociliary transport in air-liquid human models using third-harmonic generation (THG) microscopy with an optical parametric amplifier laser source at 1,300 nm. By exploiting the intrinsic nonlinear contrast at optical heterogeneities, THG provides high-resolution images of both epithelial structures and the overlying mucus layer without fluorescence staining or sample processing as recently shown for other lung-tissue environments. Time-lapse THG imaging reveals depth-dependent transport dynamics within the mucus, offering new insights into mucociliary transport. Our approach offers a physiologically relevant way to assess mucociliary function *in vitro* and could support studies on respiratory diseases, drug delivery and epithelial remodeling.

## Highlights

• THG visualizes airway epithelium and mucus layer in human-derived ALI models
• Label-free THG contrast differentiates ciliated from goblet cells
• Depth-resolved tracking reveals MCT velocity gradient across the mucus layer
• Combined THG and fluorescence monitors mucociliary recovery in CF models

## Graphical abstract

**Figure:**
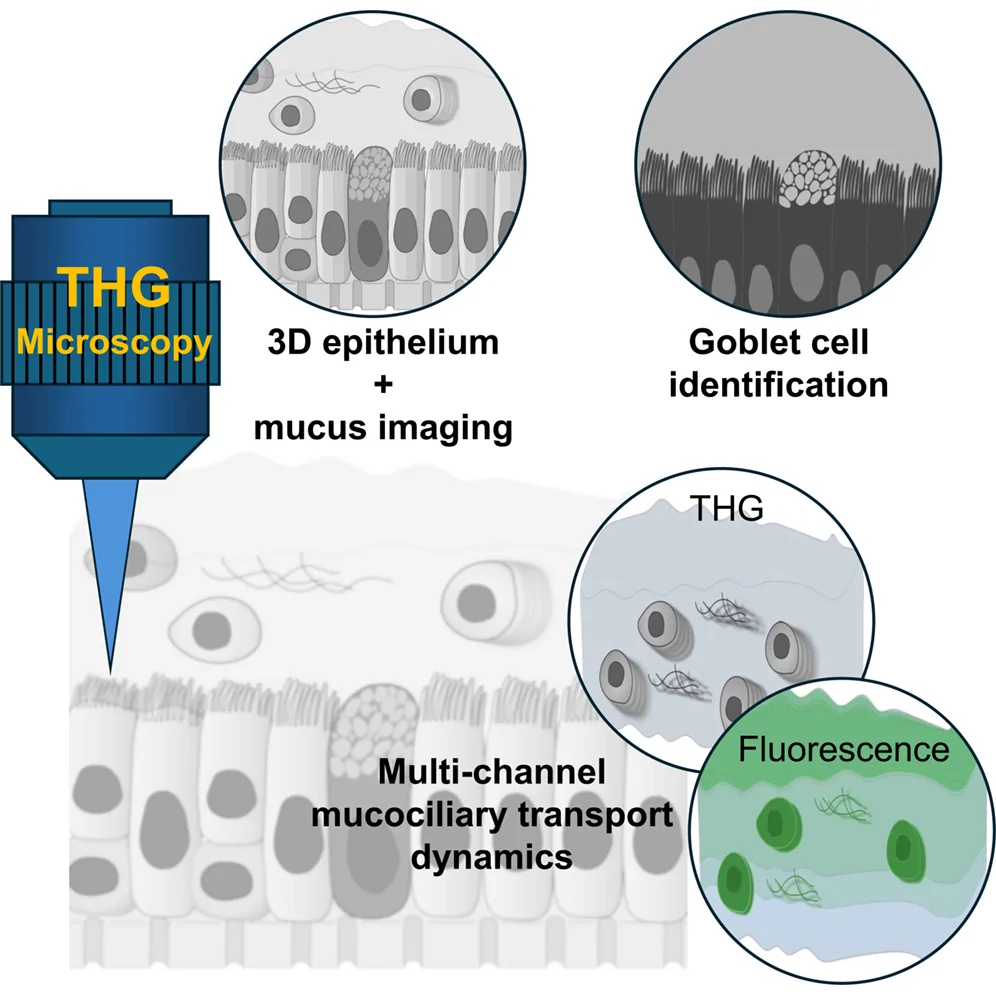

## Teaser

Tissue engineering; Cell biology

## Introduction

Human-based *in vitro* tissue models have become central to translational research because they can reproduce essential aspects of human physiology better than two-dimensional cultures or animal models, and they are aligned with regulatory trends favoring human-relevant testing approaches.^1^ When derived from patient-specific cells, these platforms enable assessment of individual response profiles, prospectively supporting personalized therapeutic strategies.^2^^,^^3^

Within the respiratory system, the airway epithelium plays a major role as the primary interface with the external environment, acting both as a physical and functional barrier. *In vitro* airway models that reproduce the epithelium architecture are therefore a valuable tool for studying infection, inflammation, and transport processes. Air-liquid interface (ALI) cultures stand out among *in vitro* systems because they differentiate into an epithelium that recapitulates key structural and functional properties and are increasingly used for the evaluation of inhaled therapeutics, for pollutant toxicity testing.^4^ Finally, their compatibility with patient-derived material make them attractive for translational studies and personalized approaches.

Mucociliary transport (MCT) is the fundamental self-clearing mechanism of the respiratory system, playing a crucial role in maintaining pulmonary health by removing inhaled pathogens and particulate matter from the airways.^5^ Impaired MCT is a hallmark of several chronic respiratory diseases, including cystic fibrosis (CF) and chronic obstructive pulmonary disease, and acts as an indicator of disease progression and therapeutic efficacy.^6^ Despite its central role in respiratory health and disease, the ability to study MCT in physiologically relevant *in vitro* models remains challenging. Existing imaging modalities often fail to combine high spatial resolution, volumetric readout, and temporal sampling compatible with ciliary and mucus dynamics, while preserving native tissue conditions.^7^ Particle-tracking approaches using fluorescent microspheres and widefield fluorescence microscopy are commonly used to quantify mucus transport,^8^^,^^9^ but they require exogenous probes that may locally perturb mucus rheology and provide limited insight into depth-resolved flow within the periciliary layer (PCL) and inner mucus regions.^10^ Optical coherence tomography (OCT) offers label-free volumetric imaging with micrometer-scale axial resolution and (especially with swept-source implementations) faster acquisition than laser scanning approaches, making it attractive for studies of mucus dynamics.^11^^,^^12^^,^^13^^,^^14^ As OCT is primarily sensitive to refractive-index discontinuities, it predominantly yields morphological contrast, which can limit its applicability in studies requiring multiplexed optical readouts, such as the integration of fluorescence channels.^15^ These considerations motivate the investigation of complementary optical approaches capable of providing simultaneous structural, dynamical, and, when necessary, molecularly sensitive readouts in ALI models, while remaining minimally invasive and compatible with longitudinal studies.

Third harmonic generation (THG) is being increasingly applied as a contrast mechanism in nonlinear microscopy thanks to its high sensitivity to optical heterogeneities and interfaces, and its complementarity with other nonlinear signals such as second harmonic generation (SHG) and multiphoton-excited fluorescence/autofluorescence (MPEF).^16^^,^^17^^,^^18^^,^^19^ Recent photonics developments have led to the availability of rugged and compact optical parametric amplifier (OPA) laser sources optimized for microscopy, which combine (i) short-wave infrared (SWIR) tunable excitation, producing THG signals in the visible range, beyond the cut-off of standard optical components and within the peak sensitivity of silicon detectors, with (ii) excitation at low-MHz repetition rates that enhance nonlinear efficiency^20^^,^^21^^,^^22^ while minimizing average power and thermal load on samples. The low-MHz repetition rate approach (1–10 MHz) has also proved successful by utilizing cost-effective, single-wavelength fiber lasers at 1,060 nm.^23^^,^^24^ Recent works have also highlighted the possibility of extending THG toward spectroscopic contrast by exploiting sample-specific resonances in combination with multicolor excitation.^25^ Owing to its sensitivity to optical discontinuities,^26^ THG is particularly well suited to label-free imaging of the air-mucus and mucus-epithelium interfaces. THG harmonic microscopy has previously been applied to lung tissue in the context of histopathological assessment and lipid body imaging, demonstrating its sensitivity to alveolar structures and tissue interfaces.^16^^,^^23^^,^^24^^,^^27^ To the best of our knowledge, its application to mucus dynamics in ALI culture models has not been previously reported.

Building on this property, here we employ THG as a label-free, subcellular-resolution modality to visualize the native mucus layer in ALI cultures using a 1 MHz OPA laser tuned to 1,300 nm. Because THG resolves individual epithelial cells and reveals fine mucus microstructure (including mucin aggregates), we show that the same contrast mechanism can be used to monitor MCT dynamics under varying conditions.

## Results

### Epithelial and mucus layer architecture

As illustrated in Figure 1A, lung epithelium is primarily composed of a columnar epithelial layer that includes goblet cells (*gob*), basal cells (*bas*), and ciliated cells (*ci*). Each ciliated cell typically possesses 200 to 300 cilia, with each cilium measuring 0.2–0.3 *μ*m in diameter and 6–7 *μ*m in length.^28^ Cilia beat at a coordinated frequency, typically of the order of 10–20 Hz, generating a synchronized, wave-like motion that drives directional MCT across the epithelial surface (Figure 1B). The epithelium surface is covered with mucus secreted by goblet cells, which traps inhaled particles and pathogens, while ciliary beating facilitates their clearance.^29^ The thickness of the mucus layer directly influences ciliary movement and transport efficiency, while abnormal mucus thickness is commonly observed in respiratory pathologies such as CF, asthma, and chronic bronchitis.^30^

**Figure 1:**
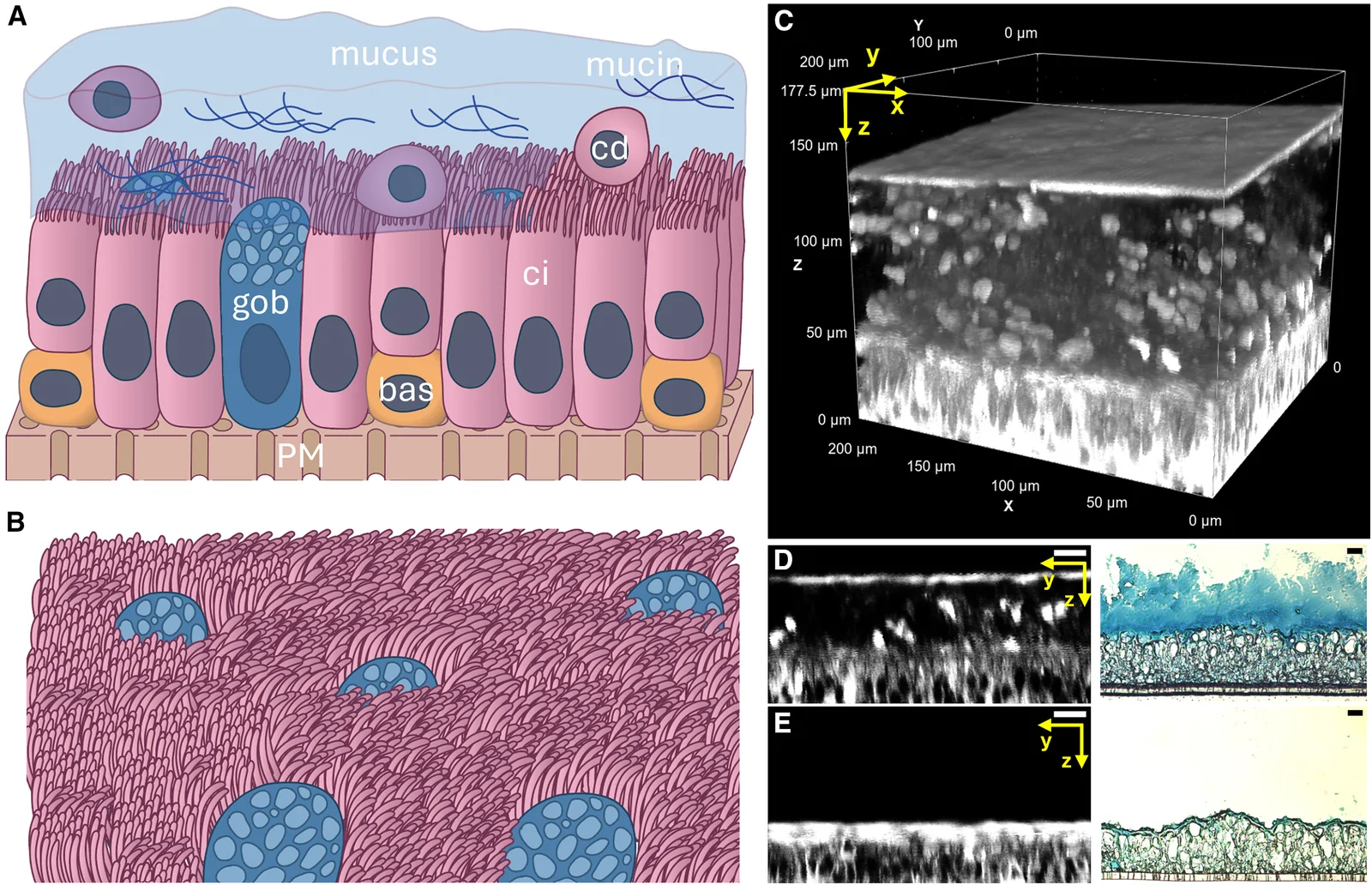
THG microscopy visualizes epithelial architecture and the overlying mucus layer (A) Cross-sectional schematic of the airway epithelium ALI model showing ci, gob, and bas on a porous membrane (PM). The apical surface is covered by a mucus layer embedding mucins and cellular debris (cd). (B) Illustration of the apical view of the epithelium showing the dense cilia on ciliated cells and the apical secretory region of goblet cells. (C) Three-dimensional THG image of an actual ALI sample showing the respiratory epithelial tissue (0–50 *μ*m) and the mucus layer (60–130 *μ*m). (D and E) Cross-sectional THG (left) and histology (right) images of airway epithelium with (D) intact and (E) removed mucus layer. Scale bars, 20 *μ*m.

To investigate the structural organization and functional dynamics of the airway epithelium, we conducted a series of THG imaging experiments on a commercial bronchial ALI model derived from cells collected after surgical polypectomy of informed and consenting patients.

Figure 1C demonstrates that both the epithelial structure and the overlying mucus layer can be simultaneously visualized by THG. Within the epithelium (*z* : [0–50] *μ*m), cell nuclei appear as signal-void regions,^31^ enabling the identification of individual cells. In the mucus region (*z* : [60–130] *μ*m), THG reveals fine structural details that can be associated with the mucin network and cellular debris (see also Figure S1). Finally, the mucus upper surface at *z* ≃ 130 *μ*m is evidenced by an extremely intense THG signal delineating a rather flat interface with air. The mucus layer thickness, from the epithelial surface to the air-mucus interface, can be estimated in a label-free fashion by THG providing a value in agreement with that obtained on cryosectioned samples stained by Alcian blue,^32^ as shown in Figure 1D.

The association of mucus with THG optical contrast is further confirmed by the bulk disappearance of the signal from this region when the mucus layer is removed by rinsing (Figure 1E). Under these conditions a strong THG signal arises from the newly generated air-epithelium surface, predominantly associated with the sharp refractive-index contrast at the epithelial surface, with a possible contribution from residual mucus remaining after rinsing. To further investigate the impact of the optical properties on cell THG contrast, we filled the apical surface of the epithelium with phosphate-buffered saline (PBS) after fixation. Under these conditions, bundles of cilia on ciliated cells became observable by THG as reported in Figure S2, which shows the same epithelial region imaged with epithelium exposed to air (Figures S2A and S2C) and after apical addition of PBS (Figures S2B and S2D). When the epithelium is fixed and immersed in PBS, cilia bundles of 6 *μ*m approximate length become distinct (inset of Figure S2D), whereas under direct air exposure, the strong THG signal from the interface with the residual mucus layer dominates, note that even in presence of PBS, the system resolution does not allow to resolve individual cilia.

In presence of an unaltered mucus layer, the upper surface of the epithelium appears as in Figure 2B. A pronounced THG signal marks regions occupied by ciliated cells. By contrast, goblet cells are revealed by the absence of THG emission. The discrimination between ciliated and goblet cells is based on their distinct THG contrast mechanisms. Ciliated cells generate strong apical THG signal due to the high density of cilia, whose membranes create optical interfaces with refractive index discontinuities that efficiently drive THG emission. Goblet cells, by contrast, appear as signal-void regions, likely because their interior is homogeneously filled with mucin granules and exhibits low refractive index contrast relative to the surrounding mucus, resulting in negligible THG signal. In the cross-sectional view (Figure 2E), goblet cells exhibit a characteristic cup-like morphology in negative contrast, whereas ciliated cells appear bright, except for their nuclei in the basal region of the epithelium.

**Figure 2:**
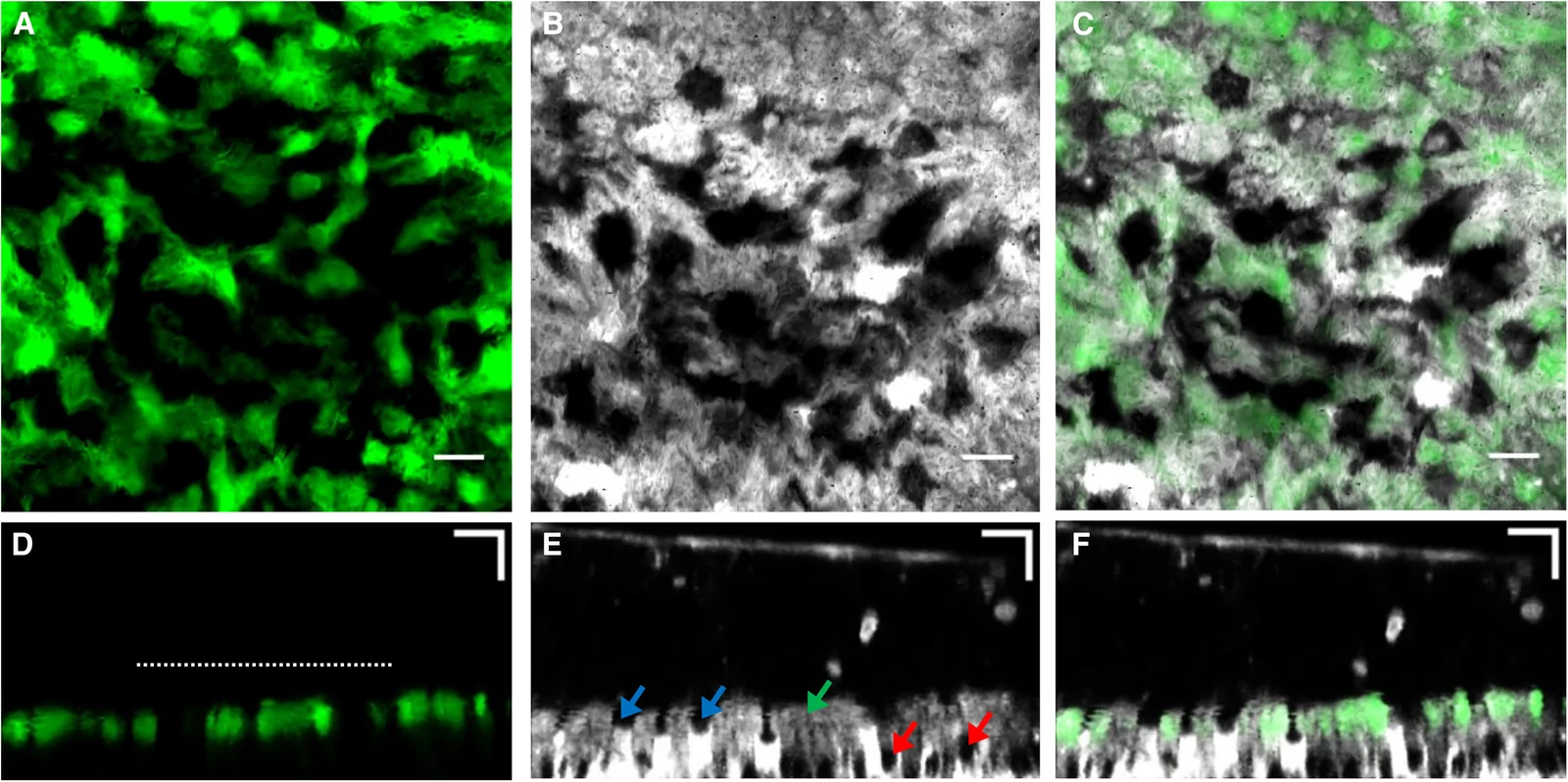
THG contrast distinguishes ciliated cells from goblet cells within the epithelium (A–C) Minimum intensity projection of (A) cilia stained with SPY650-tubulin, (B) THG signal from the cilia-mucus interface in the same region, and (C) merged image. (D–F) Examples of orthogonal views along the vertical direction. Note the larger spatial extent compared with panels (A–C). Goblet cells appear as void within the upper epithelial layer. Nuclei void signals are indicated in red, goblet cell granule void signals in blue, and the cilia-mucus interface signal in green. Scale bars, 10 *μ*m in (A–C), 20 *μ*m in (D–F).

These assignments are corroborated by simultaneously acquired SPY650-tubulin fluorescence (Figures 2A and 2D), which selectively labels cilia and shows strong correspondence with the apical regions of structures associated with ciliated cells.^33^ The ability to identify goblet cells through intrinsic nonlinear optical response (also confirmed by a control measurement performed in the absence of staining agents, as in Figure S3) provides a valuable means to assess their distribution and abundance across different tissues.

### THG-enabled monitoring of mucociliary transport

The presence of multiple THG emitting structures within the mucus layer (such as those marked with red arrows in the orthogonal view in Figure 3E) can be directly exploited for tracing mucus dynamics, as these endogenous objects move in concert with the mucus layer. Figures 3A–D show traces of their displacements over a 3.2 min time span (Video S1). The extracted traces indicate that the objects move along the same direction but have a gradient in speed moving from the PCL (Figure 3A, 55 *μ*m height) to the upper mucus region (Figure 3D, 85 *μ*m height). This height-dependent speed modulation is prominent in the time-resolved orthogonal view shown in Video S2.

**Figure 3:**
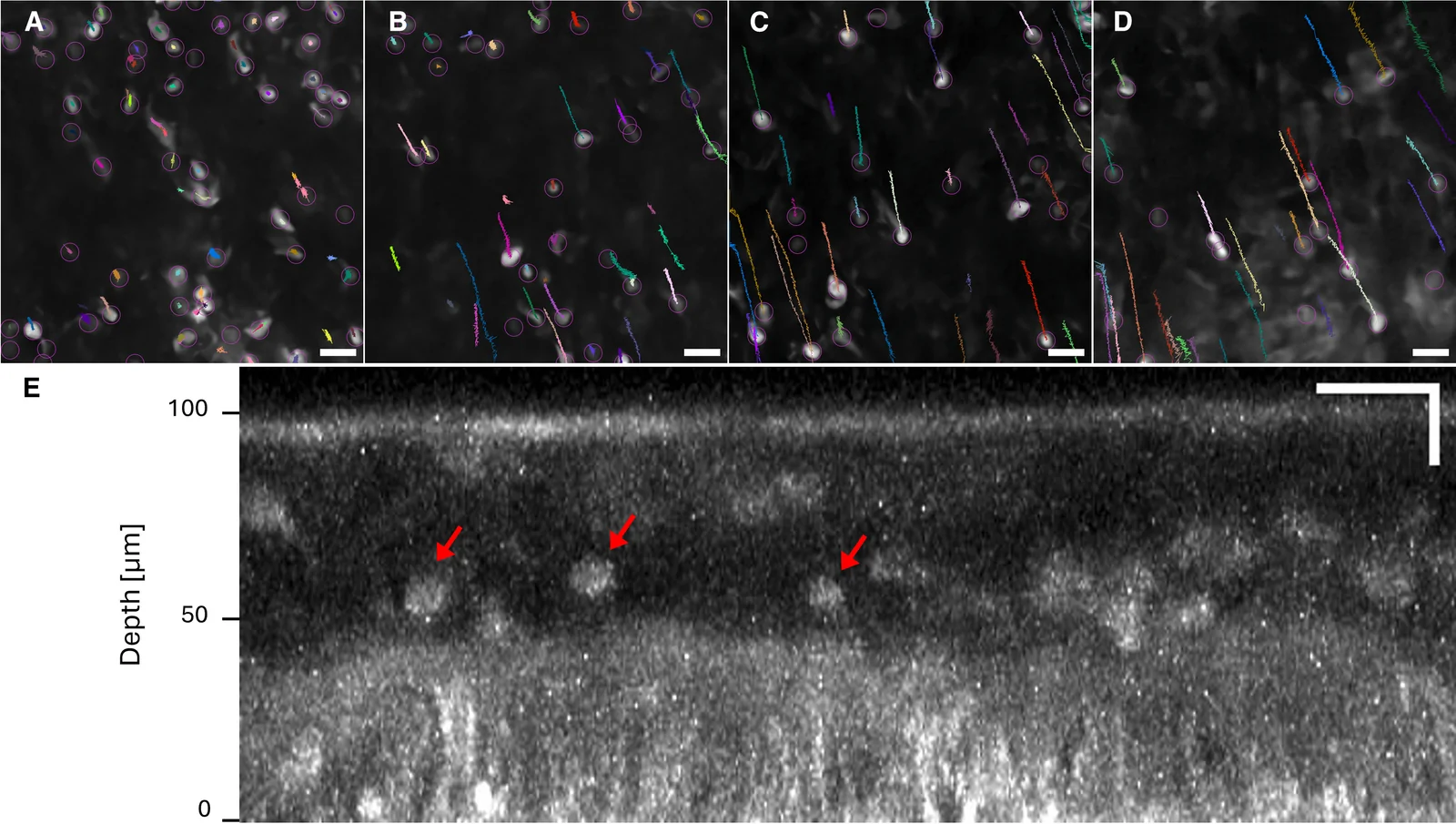
Depth-resolved tracking reveals a velocity gradient across the mucus layer (A–D) Tracking of cellular debris in the mucus at different heights above the epithelial base: (A) 55 *μ*m (near the PCL), (B) 65 *μ*m, (C) 75 *μ*m, and (D) 85 *μ*m. A time-lapse orthogonal view of the same region is shown in Video S2. (E) Maximum-intensity orthogonal view of the THG signal from the mucus and epithelium. Red arrows indicate cellular debris within the apical mucus layer. Scale bars, 20 *μ*m.

Video S1. Tracking of mucins and cellular debris in the mucus layer at different heights above the epithelial base: (A) 55 μm, (B) 65 μm, (C) 75 μm and (D) 85 μm

Video S2. Time-lapse orthogonal view showing the THG signal from mucus and epithelium, corresponding to the region depicted in Figure 3

In the dataset presented in Figure 3, we observe a slower transport velocity closer to the PCL, consistent with the vertical organization of airway mucus, comprising a low-viscosity layer adjacent to the epithelium and a denser, more viscoelastic gel-like mucus layer above. The upper mucus layer displays greater structural cohesion due to its higher mucin concentration, enabling more efficient transmission of ciliary propulsion and entraining embedded components, such as cellular debris, within the moving mucus bulk. A marked transport speed difference between the periciliary and upper layers has also been reproduced and unraveled by computational fluid dynamics models.^34^^,^^35^

The mucus transport velocities observed in this study are generally lower than those reported in the literature, which themselves span an exceptionally broad range (up to several mm/s), that can be attributed to the substantial variability in sample characteristics and conditions. In a set of measurements using samples from 3 donors, we measured transport speeds ranging from 1.2 to 2.7 *μ*m/s after averaging on multiple individual tracks obtained by following debris over several tens of seconds at a fixed height in the mucus. In some of these tracks we observed speed spikes up to 15 *μ*m/s. Although previous studies have reported that ALI models generally exhibit slower transport rates than *in vivo* tissues,^36^ and that the motion of endogenous mucus is typically reduced compared to that inferred from tracer bead tracking,^37^^,^^38^ the discrepancy observed here remains appreciable. We do not necessarily attribute this difference to sample dehydration or tissue damage, as (i) no decrease in mucus layer thickness was detected over time and (ii) the epithelial architecture remained morphologically intact throughout the measurements. The observed velocities are also not constrained by the acquisition speed of our system, in Figure 3, each 2D frame of 509 × 509 pixels (FoV, *L*^2^ =200 × 200 *μ*m^2^) was acquired with a rate of 1.55 Hz (nominal value from the microscope software) or, equivalently, a frame-to-frame interval of Δ*t* = 0.645 s. This value defines the temporal resolution of the system and sets an upper bound on measurable transport velocities. For example, considering the maximum observed speed of *v* = 15 *μ*m/s, the displacement between two successive acquired frames is *v* × Δ*t* = 9.7 *μ*m, which should ensure the ability to sample moving objects multiple times, provided that their trajectories are not too peripheral with respect to the field of view. Higher frame rates can be achieved by increasing the scanning speed provided that the sensitivity remains compatible with the measurements. Within this work, we did not further pursue absolute quantitative speed investigations in multiple samples, as the primary objective is not to establish reference values for MCT, but rather to demonstrate the imaging capabilities of the proposed THG-based approach applied to human airway epithelial models.

The label-free access to volumetric mucus dynamics enables the investigation of MCT in ALI models affected by specific respiratory conditions and their real-time response to *stimuli*. This approach is demonstrated in Figure 4 and Video S3, where increasing PBS volumes were applied to the apical surface of an epithelium derived from a CF patient. We clearly observe that CF mucus, known for being abnormally thick and viscoelastic, responds strongly to even modest dilutions. In the absence of PBS (A), mucus movement is minimal, consistent with stagnant transport. However, following PBS application, MCT velocity increased dramatically (∼11.6-fold) 6 h after application of 10 *μ*L of PBS (C). These results highlight the potential of THG-monitoring for pharmacological applications both for assessing topical therapies for respiratory diseases and for studying inhalation-based delivery routes for systemic drug absorption through the airway epithelium.

**Figure 4:**
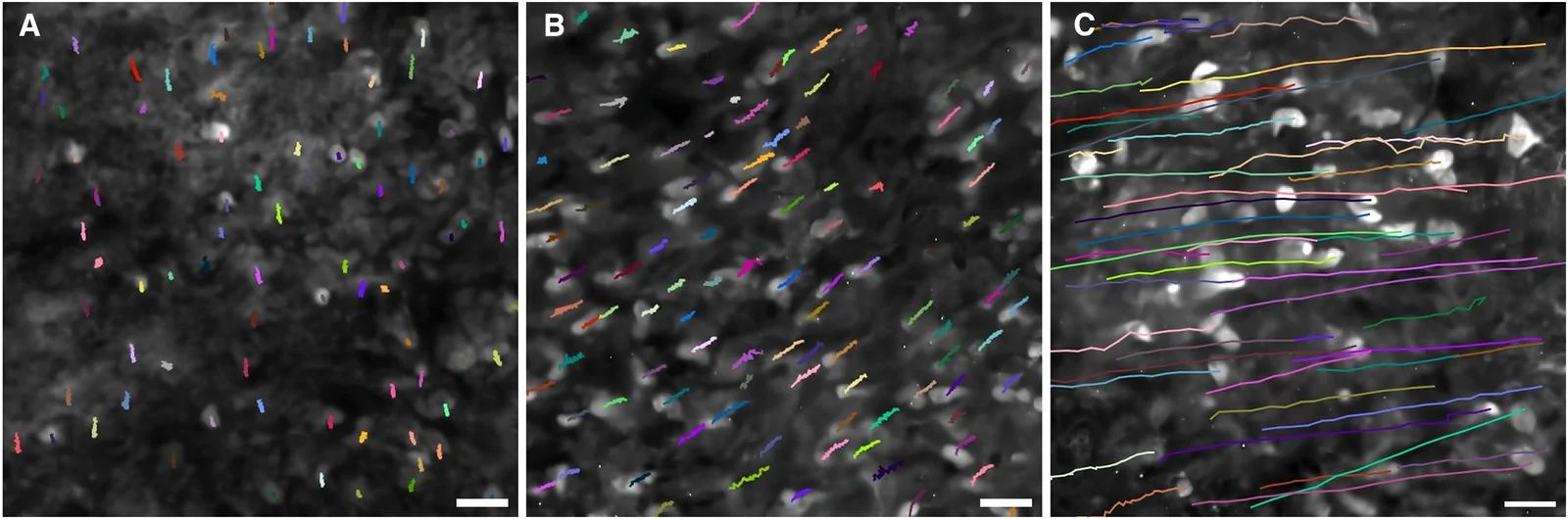
THG imaging captures the restoration of MCT in CF-epithelium upon PBS hydration Maximum intensity projection of the mucus layer of epithelial tissue acquired 6 h after PBS application. The epithelium was cultured with primary bronchial cells from a patient with CF, where (A) 0 *μ*L, (B) 5 *μ*L, and (C) 10 *μ*L of PBS were applied apically. Scale bars, 20 *μ*m.

Video S3. Maximum intensity projections of the mucus layer of epithelial tissue from CF-patient following apical application of PBS: (A) 0 μL, (B) 5 μL, and (C) 10 μL

To gain further mechanistic insight into how osmotic agents modulate MCT, we applied a 6% NaCl solution supplemented with sulfo-cyanine5 (Cy5) dye to the apical surface of a CF model with a nebulizer (Figure S4) and monitored the effect on MCT over time. As reported in Figure S5 and Video S4, the approach allows tracking the diffusion dynamics of hypertonic saline through the mucus layer and monitors the progressive restoration of MCT over time. Initially, mucus and mucins exhibit minimal movement consistent with CF conditions (A). Upon deposition of the solution (B), the hypertonic saline rapidly penetrates and diffuses through the mucus layer within 2 min, restoring MCT (C and D). MCT enhancement was observed immediately after diffusion, in agreement with the rapid MCT improvement reported upon hypertonic saline treatment in CF.^39^ While previous investigations could show that nebulized hypertonic saline enhances mucociliary clearance in CF,^40^ the nonlinear (THG and MPEF) approach demonstrated here allows simultaneous, high-resolution visualization of both NaCl diffusion and mucus movement.

Video S4. Maximum intensity projection and corresponding orthogonal views (along the dashed line) of epithelial tissue from CF patients following apical nebulization of 6% NaCl solution supplemented with Cy5 dye

These results gain further significance when compared to the widely adopted experimental approaches, where MCT is typically assessed by fluorescent bead (FB) tracking under widefield microscopy. While this method provides insights into surface-level mucus dynamics, limitations have been reported.^41^ Larger beads (typically >10 *μ*m in diameter) are often employed to ensure visibility and ease of tracking; however, their size restricts them to the mucus surface, preventing penetration into deeper layers and thereby offering limited information about inner-layer dynamics. Smaller beads (1 *μ*m or less) have the potential to probe deeper into the mucus and sample pore-scale microenvironments but they tend to be weakly coupled to large, cohesive mucus assemblies and therefore may move independently of bulk mucus transport.^37^^,^^42^ Moreover, because FB assays typically involve applying an exogenous bead suspension to the airway surface, the added liquid can locally dilute or transiently rehydrate the mucus layer, altering its concentration and rheological properties and transiently biasing MCT measurements.^43^ As a benchmark experiment, 1 *μ*m FBs were applied to the mucus surface and imaged 2 h later (Video S5). Prior to bead application, the mucus exhibited largely unidirectional transport (A). Following bead deposition, both FBs and endogenous mucus components displayed more disorganized, multidirectional motion (B). This temporary disruption likely arises from the bead application procedure itself, rather than a persistent alteration of mucociliary function. Indeed, a pre-post analysis (Video S6) shows that approximately 20 h after bead application, MCT stabilizes and recovers its unidirectional character. A similar behavior was observed on *N* = 3 samples.

Video S5. (A) Maximum intensity projection of MucilAir prior to the application of FBs; (B) 2 h post-application

Video S6. (A) Maximum intensity projection of MucilAir prior to the application of FBs; (B) 20 h post-application

## Discussion

THG microscopy is a powerful nonlinear imaging modality, highly sensitive to optical interfaces and microscopic heterogeneities.^16^^,^^20^ Its application to physiologically sensitive human-derived systems has recently become increasingly viable with the advent of tunable femtosecond OPAs and single-wavelength fiber lasers operating at low-MHz repetition rates.^20^^,^^21^^,^^22^^,^^23^^,^^27^^,^^44^ By concentrating excitation energy into fewer pulses, these sources deliver the high peak powers required to drive higher-order nonlinear processes while maintaining modest average power, in contrast to conventional titanium-sapphire and optical parametric oscillator (OPO) lasers operating at ∼80 MHz. At the same time, OPAs remain compatible with microsecond dwell time scanning regimes, ensuring the possibility of acquiring volumetric imaging of dynamical systems. The advantage of the low-MHz excitation regime is demonstrated in Figure S6 and Video S7, where an ALI model was imaged using both an 80 MHz OPO laser and the 1 MHz OPA system. The OPO required an average power of 36.0 mW to generate sufficient THG contrast for qualitative comparison with the OPA operating at 2.1 mW.^45^ As shown, the use of the 80 MHz system led to a significant reduction of the mucus layer thickness over 21 min, most likely due to evaporation. Morphological changes in the epithelium and the appearance of damage-associated emission were also observed.

Video S7. Maximum intensity projection and orthogonal view of lung epithelial tissue sequentially scanned under a 1 MHz OPA source (left) and an 80 MHz OPO source (right) over the same epithelial region over 21 min

THG imaging applied to ALI *in vitro* models of human airway epithelium provides direct access to structural and dynamical information without the need to process the samples or introduce exogenous agents. This represents a clear advantage, as it preserves the system in conditions close to its natural state and enables longitudinal measurements over time with minimal perturbation. We show that THG contrast allows clear delineation and quantification of the mucus layer thickness covering the epithelium. Within the epithelial layer, characteristic THG morphological features permit discrimination among different cell types. Furthermore, by tracking the position of objects naturally present within the mucus, it is possible to monitor mucus transport as a function of three-dimensional localization, with micrometer spatial resolution.

Notably, THG imaging can be readily combined with complementary optical readouts to extend its range of applications. Its ability to track MCT dynamics under external *stimuli* makes it a valuable tool for drug screening, when combined with fluorescently labeled compounds.^46^^,^^47^ Similar multi-channel strategies can be applied to investigate epithelial remodeling (via SHG to monitor fibrillar collagen)^48^ and mucus abnormalities in disease models such as CF and chronic bronchitis. The potential of THG for short- and long-term monitoring of human 3D airway models is particularly compelling given current regulatory shifts toward human-based systems in preclinical and drug development studies, in full ethical alignment with the 3 R principles advocating the reduction and refinement of animal experimentation.^49^

### Limitations of the study

As noted in the manuscript, the primary limitation of our approach stems from its raster-scanning operation, which limits acquisition speed. Sufficient imaging speed is critical for resolving mucus dynamics, minimizing motion artifacts, and ensuring accurate tracking. In practice, temporal resolution is limited not only by measurement sensitivity and scanner performance, but ultimately by the fundamental trade-off between dwell time and laser repetition rate, as at least one pulse per scanning position is required.

While the replicates per condition adequately support this proof-of-concept study, larger sample sizes will be required to comprehensively evaluate MCT dynamics across healthy and CF phenotypes.

## Resource availability

### Lead contact

Requests for further information and resources should be directed to and will be fulfilled by the lead contact, Luigi Bonacina (luigi.bonacina@unige.ch).

### Materials availability

This study did not generate new materials.

### Data and code availability

• The data that support the findings (microscopy images in .tiff format) of this study are available on the data repository Yareta. The DOI is listed in the key resources table.
• The customized code for orthogonal projection script is available on the data repository Yareta. The DOI is listed in the key resources table.
• Any additional information required to reanalyze the data reported in this study are available from the lead contact upon request.

## Acknowledgments

We gratefully acknowledge the funding from the European Project FAIR CHARM (H2020 LEIT 10.13039/100011273Information and Communication Technologies 101016457; https://www.faircharm.eu).

## Author contributions

Conceptualization, D.K., A.L., M.A., and L.B.; methodology, D.K.; investigation, D.K., A.L., and M.B.; formal analysis, D.K.; writing – original draft, D.K.; writing – review and editing, D.K., A.L., M.A., P.V.B., and L.B.; funding acquisition, P.V.B. and L.B.; resources, S.H. and S.C.; supervision, L.B.

## Declaration of interests

Authors S.C. and S.H. are co-founders of Epithelix Sàrl and currently employed as CEO and CSO. The company provided the samples used in this study under a research agreement.

## Declaration of generative AI and AI-assisted technologies in the writing process

During the preparation of this work, the authors used Claude (Anthropic) and Gemini (Alphabet) in order to assist with refining sentence phrasing and improving clarity in select passages of the text. After using these tools, the authors reviewed and edited the content as needed and take full responsibility for the content of the publication.

## STAR★Methods

### Key resources table

**Table:** 

REAGENT or RESOURCE	SOURCE	IDENTIFIER
**Biological samples**		

MucilAir™ human bronchial ALI cultures (donors A–L)	Epithelix Sàrl	N/A
CF-MucilAir™ human bronchial ALI cultures (donors M, N; ΔF508)	Epithelix Sàrl	N/A

**Chemicals, peptides, and recombinant proteins**		

SPY650-Tubulin	Spirochrome	Cat#SC503
Sulfo-Cyanine5 carboxylic acid	Lumiprobe	Cat#13390
Alcian blue	Sigma-Aldrich	Cat#B8438
Fluorescent microspheres, 1 μm	Sigma-Aldrich	Cat#L4655-1 ML
Hypertonic saline, 6% NaCl (MucoClear)	PARI	Cat#077G3000
OCT compound	Cellpath	Cat#KMA-0100-00 A
MucilAir culture medium	Epithelix Sàrl	Cat#EP04MM
Paraformaldehyde solution 36.5% in water	Sigma-Aldrich	47608-250 ML-F

**Software and algorithms**		

Fiji	Schindelin et al.^50^	RRID: SCR_002285
TrackMate (Fiji plugin)	Ershov et al.^51^	https://imagej.net/plugins/trackmate/
NIS-Elements Advanced Research	Nikon	RRID: SCR_027181
IgorPro	WaveMetrics	RRID: SCR_000325
Custom Python orthogonal-projection scripts	Yareta data repository	https://doi.org/10.26037/yareta:s4p3fw376jcjhe26lv4gteakt4

**Deposited data**		

Tiff files for fluorescence channel and denoised images of Figure 2	Yareta data repository	https://doi.org/10.26037/yareta:qn3ia23wnbgdfh7pbk4rghe6xi

**Other**		

CRONUS-3P OPA laser (1 MHz, 1250–1800 nm)	Light Conversion	https://lightcon.com/products/cronus-3p-femtosecond-lasers-for-advanced-nonlinear-microscopy/
InSight X3 OPO laser (80 MHz, 680–1300 nm)	Newport/Spectra-Physics	https://www.spectra-physics.com/en/f/insight-x3-tunable-laser
TriM Scope Matrix microscope	Miltenyi Biotec	N/A
XLPLN25XWMP2 objective (25×, NA 1.05, WD 2 mm)	Olympus	N/A
GaAsP-photocathode PMT	Hamamatsu	Cat#H12056-40
Multialkali PMT (IR-extended)	Hamamatsu	Cat#H12056-20
Mini-incubator	Okolab	Cat#H501-T
Nebulizer	Omron	Cat#NE-U100
Thermal power sensor/meter	Ophir	3 A-PF-12; StarBright
Transwell inserts (6.5 mm)	Oxyphen	N/A
Cryostat	Leica	CM3050 S

### Experimental model and study participant details

#### Human 3D lung epithelial models

Commercially available primary human airway epithelial cultures (MucilAir™, Epithelix Sàrl, Geneva, Switzerland) were used in this study. These cells were obtained from patients undergoing surgical polypectomy. All experimental procedures were fully explained to participants, and informed consent was obtained from all donors. The study was conducted in accordance with the Declaration of Helsinki (Hong Kong amendment, 1989) and received approval from the *Commission Cantonale d’Éthique de la Recherche Scientifique de Genève* (reference number 15–062). All samples were collected with informed consent as part of ethically reviewed and approved protocols. Collection procedures included (i) obtaining detailed donor medical histories to ensure the safety of both donors and researchers, and (ii) approval by an Institutional Review Board (IRB).

The donor information for each experiment is given in Table S1 in supplemental information. Donor A was a 61-year-old female, African American and non-smoker, donor B was a 22-year-old male, Caucasian, non-smoker. Donor C was a 22 years old male, Caucasian, non-smoker and donor D was a 59-year-old male, Caucasian, non-smoker and donor E was a 27-year-old male, Caucasian, non-smoker. Donor F was 35-year-old male, origin not reported, non-smoker and donor G was 59-year-old female, Caucasian, non-smoker. Donor H was a 22-year-old male, Caucasian, non-smoker. Donor I was a 72-year-old male, Hispanic, non-smoker. Donor J was a 72-year-old male, Hispanic, non-smoker. Details for donor K were not reported. Donor L was a 59-year-old female, Caucasian, non-smoker. Donor M was a 30-year-old female, origin not reported, non-smoker and had Homozygote ΔF508 mutation which is the most common mutation causing CF. Donor N was a 39-year-old male, origin not reported, non-smoker and had ΔF508 mutation.

Donors of both sexes were included in this study (Table S1). As the aim of this work was to demonstrate the imaging capabilities of the THG-based approach rather than to establish reference physiological values, the study was not designed to assess the influence of sex or gender on the reported measurements.

#### Culture conditions

Primary human bronchial epithelial cells were isolated from biopsies provided by consenting donors. The epithelial cells were cultured on the microporous membrane of a Transwell insert (Oxyphen, Wetzikon), and after several days, were transitioned to ALI conditions for at least 28 days to allow full differentiation into a ciliated epithelium. Basolateral medium (EP04MM, Epithelix Sàrl; 700 *μ*L) was replenished twice weekly. Throughout the culture period, inserts were maintained at 37°C in a 5% CO_2_ humidified atmosphere and handled strictly under sterile conditions. During image acquisition, the tissue was maintained at 37°C in a mini incubator (H501-T, Okolab), connected to a humidifying pump to ensure a stable environment. Each imaging session lasted no longer than 5 h, after which the tissue was discarded to prevent any risk of contamination.

### Method details

#### Nonlinear microscopy

The THG signal from epithelial tissue was generated at 1300 nm using the Short-Wave Infrared Microscope (SWIM) at the University of Geneva. SWIM combines a TriM Scope Matrix with custom SWIR-optimized optics (Miltenyi Biotec, Bielefeld, Germany) and a CRONUS-3P laser (1 MHz OPA, 1250–1800 nm; Light Conversion, Vilnius, Lithuania). The experimental configuration and sample placement are illustrated in Figure S7. The system was equipped with an Olympus XLPLN25XWMP2 water-immersion objective (25×, NA 1.05, WD 2 mm).

THG emission was detected using a GaAsP-photocathode PMT (Hamamatsu H12056-40) with a 450/60 nm bandpass filter. Fluorescent signal was collected with an infrared-extended multialkali-photocathode PMT (Hamamatsu H12056-20) with a 650/50 nm bandpass filter (SPY650-tubulin, Cy5 emission), and a GaAsP-photocathode PMT (Hamamatsu H12056-40) with a 525/50 nm bandpass filter (fluorescent beads).

The nominal pulse duration of the CRONUS-3P at 1300 nm is 50 fs at the laser output. Group delay dispersion (GDD) pre-compensation was optimized by maximizing the SHG and THG signals from inorganic nanoparticles^52^ dispersed on a microscope slide, yielding an optimal setting of −3.6 kfs^2^. A telescope was used to expand the CRONUS-3P beam to overfill the back aperture of the objective to maximize effective NA.

By inspecting datasets from samples containing fluorescent beads with a nominal diameter of 1 *μ*m suspended in mucus (similar to the conditions of Videos S5 and S6), we systematically observed objects exceeding 1 *μ*m in the *xy* plane, as reported in Figures S8C and S8D. This observation provides a conservative upper estimate for the effective lateral resolution achieved under realistic measurement conditions within the sample. The axial profile is affected by the complex interplay between volumetric acquisition speed and mucus transport dynamics, as demonstrated by the *xz* orthogonal view in Figure S8, where the effect of transport along the *x* direction becomes evident during full volumetric acquisition.

For the comparison shown in Figure S6, an InSight ×3 laser set to 1300 nm (80 MHz OPO; ∼130 fs pulse duration at laser output at 1300 nm, assuming transform-limited Gaussian pulses; Newport/Spectra-Physics, Milpitas, CA, USA) was aligned through the same microscope platform. No additional beam expansion or external dispersion optimization was performed for the InSight source during these measurements. The average power was measured after the microscope objective (without immersion liquid) using an Ophir 3 A-PF-12 thermal sensor and StarBright meter.

#### Imaging acquisition parameters

Three-dimensional images were obtained by z-stacking a 2D collection of consecutive scans with 1064 × 1064 pixels and a step size of 0.5 *μ*m (Figures 1 and S1–S3) and 1 *μ*m (Figure 2). For time-lapse imaging, three-dimensional data was captured with 509 × 509 pixels and a frame time of 0.644 s with a step size of 1 *μ*m (Figures 3 and 4), a step size of 2 *μ*m (Video S5), a step size of 3 *μ*m (Figure S6, Video S6) and a step size of 4 *μ*m (Figure S5).

#### Histology and mucus removal

THG images are compared with histology sections of adjacent tissue regions. For mucus removal, 20 *μ*L of 0.9% NaCl solution was added to the apical side of the inserts, followed by incubation at 37°C for 20 min. The solution was then entirely removed after thorough mixing with a pipette. Subsequently, the inserts were fixed by complete immersion in 4% paraformaldehyde (PFA) in PBS for 20 min at room temperature. The remaining inserts were fixed by immersing the basolateral side in 1 mL of 4% PFA for 20 min, without adding any medium to the apical side to preserve the native mucus layer. After fixation, tissues were embedded in OCT compound (Cellpath, KMA-0100-00 A) and immediately frozen at −20°C. Sections were then cut at 10 *μ*m thickness using a cryostat (Leica CM3050 S) and mounted onto glass microscope slides. The microscope slides were subsequently stained with Alcian blue (Sigma-Aldrich, B8438) for 10 min, then thoroughly washed and mounted.

#### Cilia labeling

SPY650-Tubulin (Spirochrome SC503) was diluted 1:1000 in media and added to the basolateral side of MucilAir, followed by a 48-h incubation before imaging.

#### Mucus transport speed analysis

Mucus transport speed analysis was conducted on samples derived from donors A, B, and D.

#### Hypertonic saline nebulization

Sulfo-Cyanine5 carboxylic acid (13390, Lumiprobe) was diluted in 6% NaCl (Mucoclear 6%, PARI) to a final concentration of 1 *μ*g/mL, then nebulized and applied to the apical side of the MucilAir insert using a nebulizer (NE-U100, Omron). The configuration of the nebulization setup is shown in Figure S4 of the supplemental information.

#### Fluorescent bead

Fluorescent beads with a mean particle size of 1 *μ*m (L4655-1 ML, Sigma Aldrich) were diluted 1:500 in PBS before being applied to the MucilAir insert. A volume of 3 *μ*L of the diluted solution was deposited onto the insert using a pipette, then the insert was kept in the incubator at 37°C and 5% CO_2_. Donors J and K were additionally included in the fluorescent bead assay for 2 h monitoring, and donors K and L were additionally included for the 24 h recovery monitoring.

### Quantification and statistical analysis

#### Image processing and projections

Z-height and xy drift were corrected in Video S4, as mechanical vibrations and movements were caused by the nebulizer positioned on the stage when operated. Image denoising for Figures 1, 2, 3A–3D, and S1–S3 was performed using Nikon NIS-Elements software. In Figures 1, 2, S2, and S3, maximum intensity projections and orthogonal views were generated using NIS-Elements software (Nikon). In Figures 3E, 4, S1, S5, and S6, maximum intensity projections and orthogonal views were generated using Fiji.^50^ For Figures 3, S5, and S6, orthogonal views were obtained by maximum-intensity projection within a defined ROI along one lateral dimension for each time point and z-slice using a custom-written code in Python. For Figure S8, orthogonal views were extracted directly from the 3D image stack using IgorPro.

#### Particle tracking

Cellular debris were tracked using the Fiji plugin *TrackMate*.^51^ Depending on image quality and object appearance, different tracking parameters were applied. The built-in *Laplacian of Gaussian* (LoG) detector was used with estimated object diameters ranging from 5 to 15 *μ*m and quality thresholds between 5 and 15. In some cases, manual tracking was performed to ensure accuracy (Videos S1, S3, S5, and S6). The effective scanning speed used for velocity calculations was extracted directly from the microscope acquisition settings.

## Supplemental information

Document S1. Figures S1–S8 and Table S1

Video S8. Brightfield video of the scanned region shown in Figure S6 and its nearby area where the scanned region (white dashed line) and its adjacent area (red dashed line) exhibit no ciliary beating after 80 MHz OPA scanning
